# Dr. Henry S. Ellwood

**Published:** 1906-04

**Authors:** 


Dr. Henry S. Ellwood, of Buffalo, died at his home in this city March 22, 1906, aged 68 years. He was born in London, Canada in 1838 and came to Buffalo about 45 years ago. He graduated in medicine at the University of Buffalo in 1868. He established a pharmacy at No. 9 West Mohawk Street which he conducted nearly forty years. Dr. Ellwood was a man of kindly nature and was noted for his generous acts of charity. He is survived by his wife, Esther E. and his son, Dr. Grant T, Ellwood.

